# Supplementary material for: Waste milk humification product can be used as a slow release nano-fertilizer
Source: Nat Commun. 2024 Jan 2;15:128. doi: 10.1038/s41467-023-44422-5 (PMC10761720; doi:10.1038/s41467-023-44422-5)
Supplement: Supplementary file 1 — Supplementary Information [file 41467_2023_44422_MOESM1_ESM.pdf]

Waste milk humification product can be used as a slow release nano-fertilizer

Yanping Zhu [a], Yuxuan Cao [a], Bingbing Fu [a], Chengjin Wang [b], Shihu Shu [a], Pengjin Zhu [c], Dongfang Wang [a], He Xu [a], Naiqin Zhong [d], Dongqing Cai [a*]

[a] *College of Environmental Science and Engineering, Donghua University, Shanghai 201620, People's Republic of China*

[b] *Department of Civil Engineering, University of Manitoba, Winnipeg, Manitoba R3T 5V6, Canada*

[c] *Guangxi Subtropical Crops Research Institute, Nanning 530000, People's Republic of China*

[d] *Institute of Microbiology, Chinese Academy of Sciences, Beijing 100101, People's Republic of China*

*Corresponding author: Dongqing Cai

Email address: dqcai@dhu.edu.cn

Lists of Captions

Fig. S1 UV spectra, optimization of treatment conditions during humification of WM: (A) dosage, (B, C) ratio and (D) time. 4

Fig. S2 3D-EEM results of treated WM at different dosages of PS and KOH with the molar ration of 1:1. 5

Fig. S3 XPS spectra of N1s for (A) WM and (B) product. 6

Fig. S4 Time-dependent variation of $S_2O_8^{2-}$ concentration during the process. Conditions: 4 g PS and 4 g KOH in 50 mL WM. 7

Fig. S5 3D-EEM results of WM added with 10 mol/L (A) EtOH and (B) TBA……………………..8

Fig. S6 Standard curves of FLA and SHA at different pHs...9

Fig. S7 Digital photographs of solutions during FLA and HLA slow release at pH 7 and 3..10

Fig. S8 Plots of different kinetic models for FLA release from SRNFF at (A) pH 7 and (B) 3. 11

Fig. S9 Set-up of pot experiment..12

Fig. **S10** Plant growth results in pot experiments...13

Fig. S11 3D-EEM variation of the product within 12 d. 14

Fig. S12 Color changes of the system during 60 min humification of 50 mL WM by 4 g KOH/ 4 g PS. 22

Table S1 Percent fluorescence response ($P_{region\ number}$) in 3D-EEM five regions of treated WM under different humification conditions based on FRI method. 15

Table S2 Determination of FLA, SHA, and HLA contents according to eqs. (6-9)....16

Table S3 Total organic carbon variation during humification....................................17

Table S4 Soil physicochemical indexs in different groups 18

Table S5 Brief comparison of carbon loss and energy consumption between this technology with conventional composting 19
Table S6 m_1 and SHA amounts at varied pHs 23
Note S1 Description of different kinetic models 20
Note S2 Quantitative analysis of 3D-EEM results 21
Note S3 Maillard reaction evidences according to color, EEM and UV spectra…….22
Note S4 Moisture, FLA and SHA loadings of SRNFF calculations…………………23
References………………………………………………………………………...24

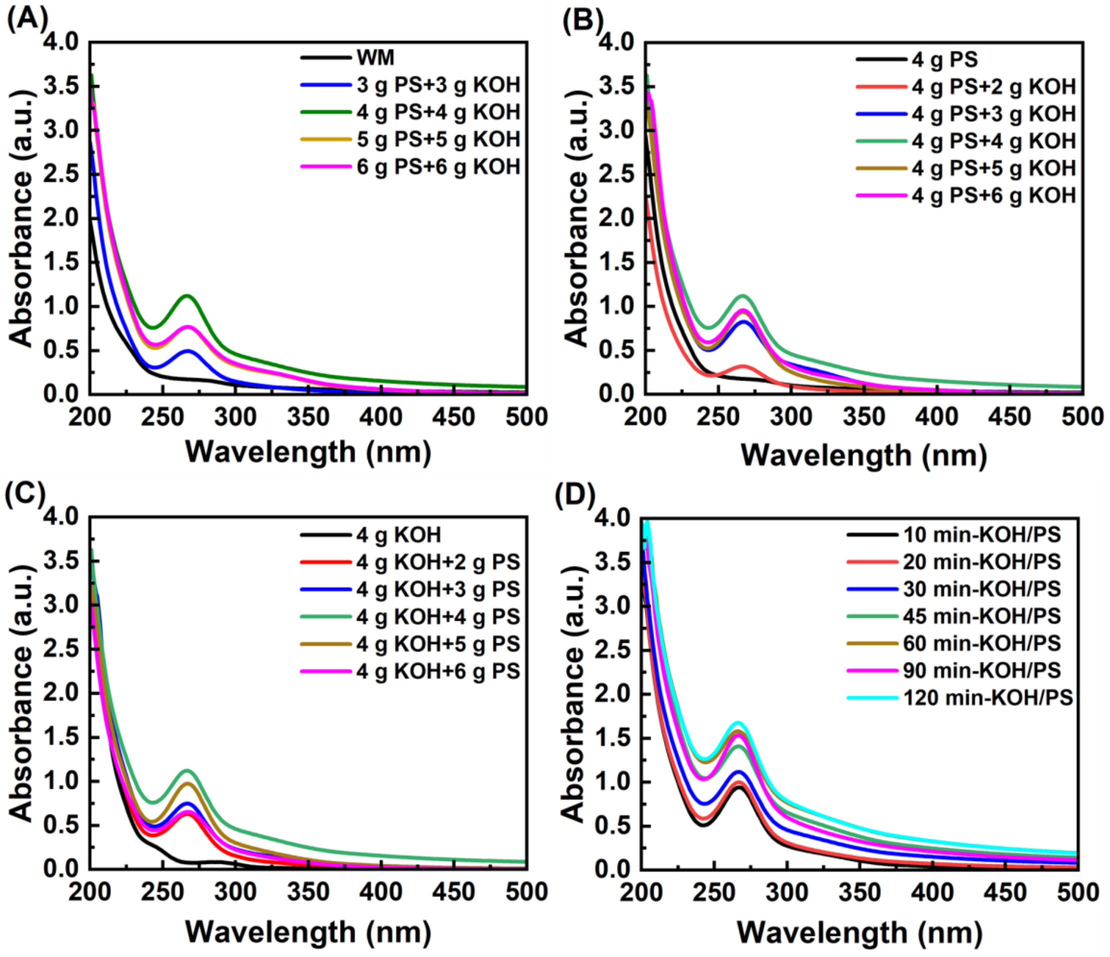

Fig. S1 UV spectra, optimization of treatment conditions during humification of WM: (A) dosage, (B, C) ratio and (D) time. Experimental conditions: WM volume=50 mL, PS or KOH dosage=0-6 g, ambient temperature=22±1 ^{o}C, and reaction time=0-2 h.

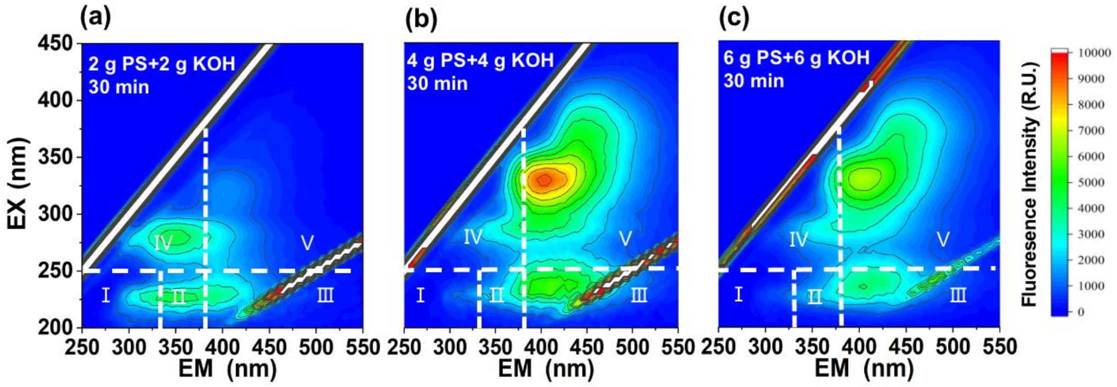

Fig. S2 3D-EEM results of treated WM at different dosages of PS and KOH with the molar ration of 1:1. Experimental conditions: ambient temperature=22±1 ºC, reaction time=30 min.

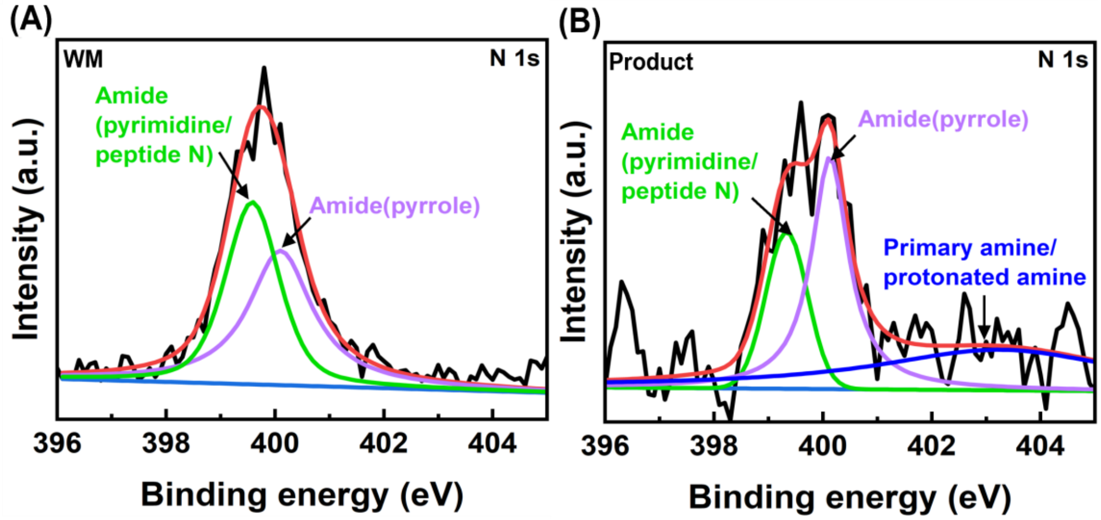

Fig. S3 XPS spectra of N1s for (A) WM and (B) product. Humification conditions: $[PS]_0$=80 g/L, $[KOH]_0$=80 g/L, ambient temperature=22±1 ºC, reaction time=1 h.

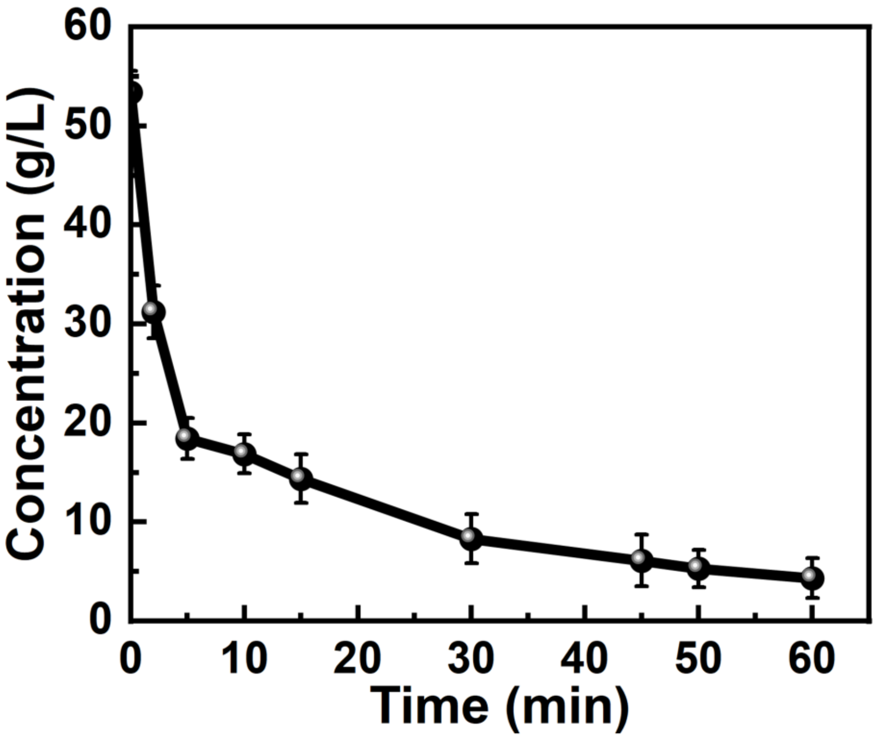

Fig. S4 Time-dependent variation of $S_2O_8^{2-}$ concentration during the process. Experimental conditions: 4 g PS and 4 g KOH in 50 mL WM, ambient temperature=22±1 ºC. Error bars represent the standard deviations from triplicate tests.

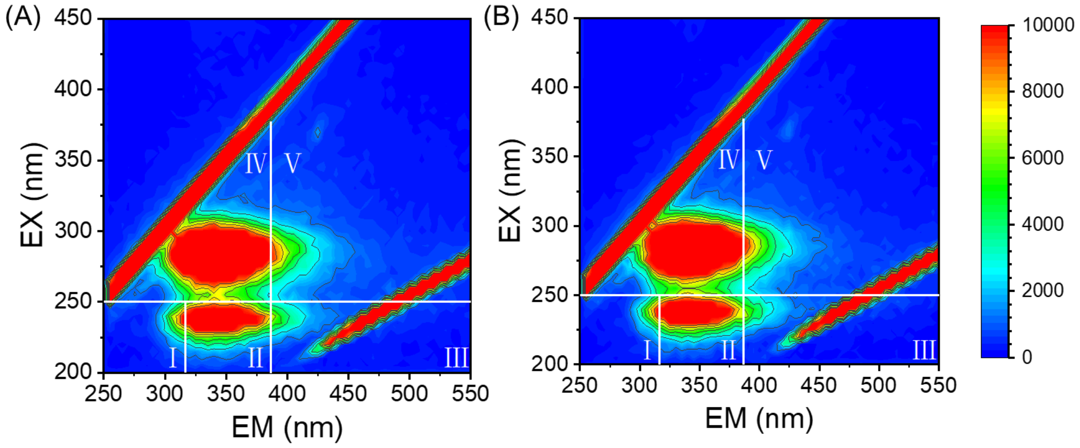

Fig. S5 3D-EEM results of 50 mL WM added with 10 mol/L (A) EtOH and (B) TBA. Experimental conditions: ambient temperature=22±1 °C, reaction time=30 min.

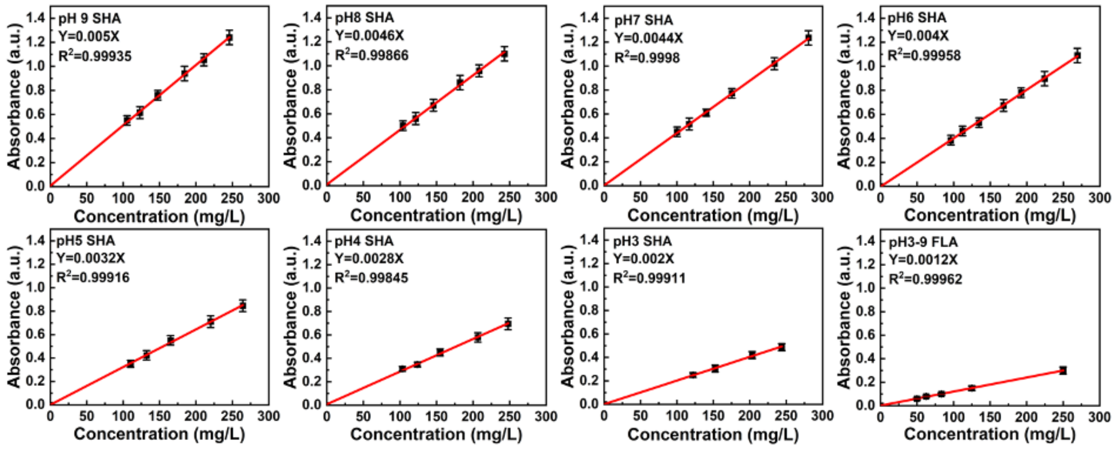

Fig. S6 Standard curves of FLA and SHA at different pHs. Error bars represent the standard deviations from triplicate tests.

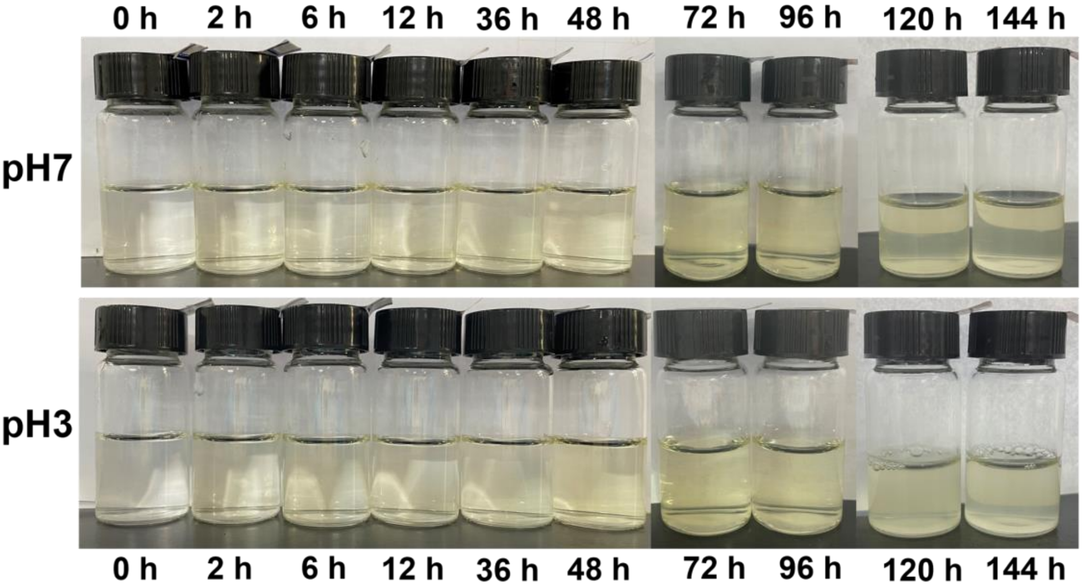

Fig. S7 Digital photographs of solutions during FLA and HLA slow release at pH 7 and 3. Experimental conditions: $[SRNFF]_0$=30 g/L , ambient temperature=22±1 ºC.

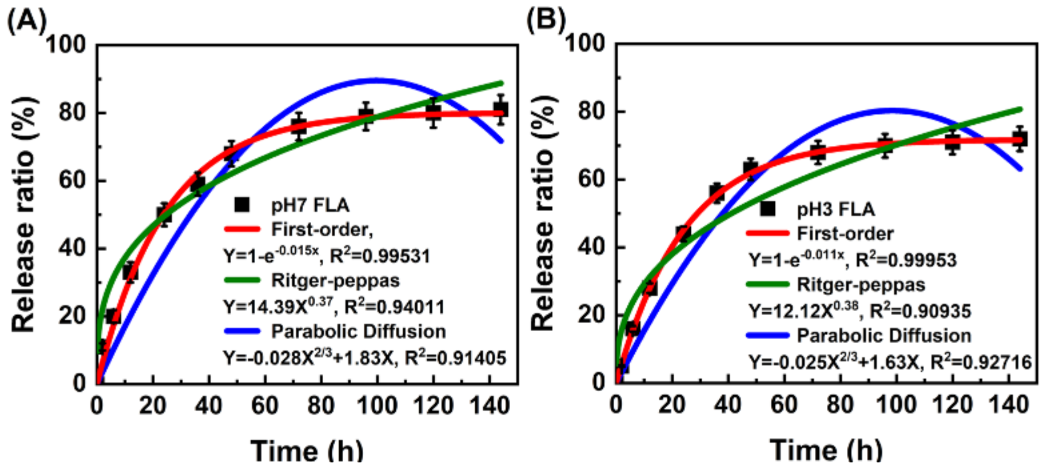

Fig. S8 Plots of different kinetic models for FLA release from SRNFF at (A) pH 7 and (B) 3. Experimental conditions: $[SRNFF]_0$=30 g/L and ambient temperature=22±1 ºC. Error bars represent the standard deviations from triplicate tests.

Groups	WM	Blank	ATP	K_2SO_4	COF	Product	SRNFF
Dosages	1.4 mL	Soil alone	0.7 g	0.14 g	1.5 g	1.4 mL	1.5 g

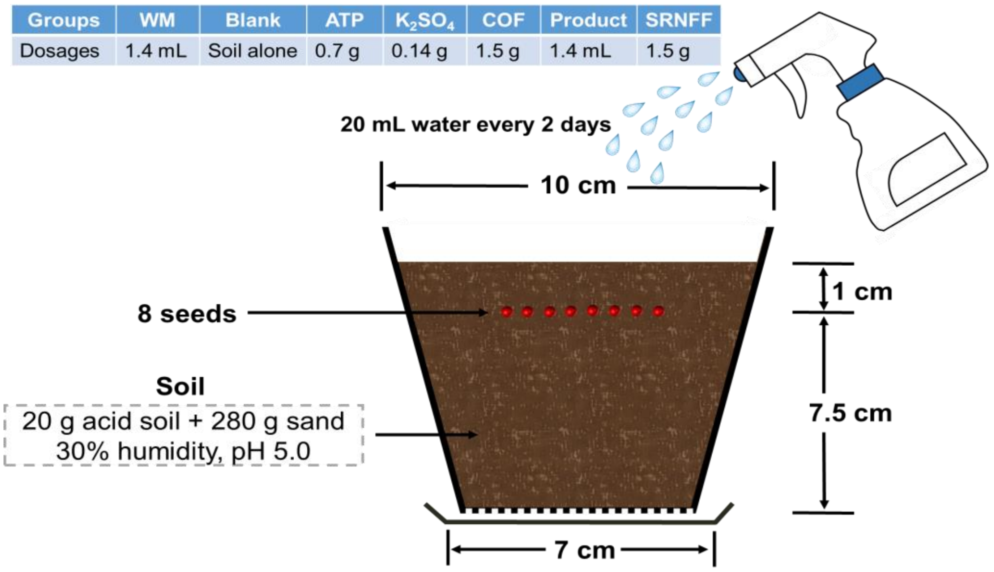

Fig. S9 Set-up of pot experiment.

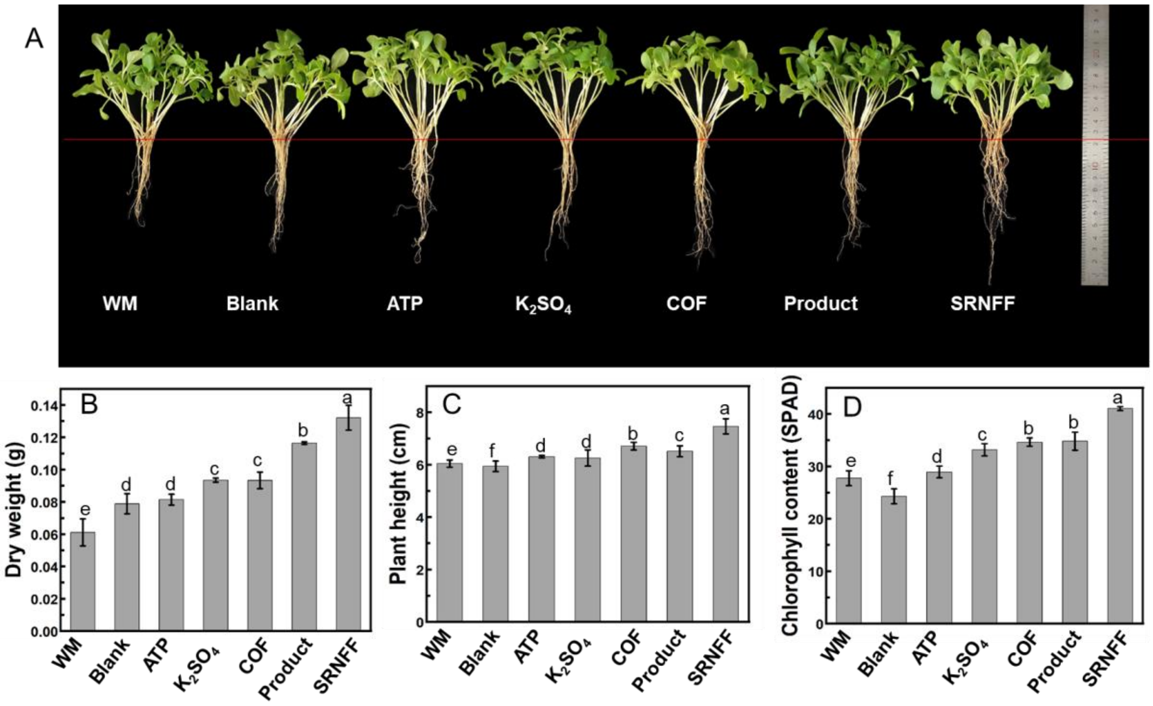

Fig. S10 (A) Overall plants photographs of each group, (B) dry weight, (C) plant height, and (D) leaves chlorophyll content of chickweeds in 21 d pot experiment with different treatments: WM, Blank, ATP, K_2SO_4, COF, Product, and SRNFF refer to 400 g soil+1.4 mL WM, 400 g soil alone, 400 g soil+0.7 g ATP, 400 g soil+0.14 g K2SO4, 400 g soil+1.5 g COF, 400 g soil+1.4 mL Product, and 400 g soil+1.5 g SRNFF, respectively. Each pot contained 8 chickweed seeds. Each group was in triplicates and error bars represent the standard deviations. Comparison among results of different treatments were via one-way ANOVA analysis, and different letters refer to significant differences with Tukey's t test at $p<0.05$.

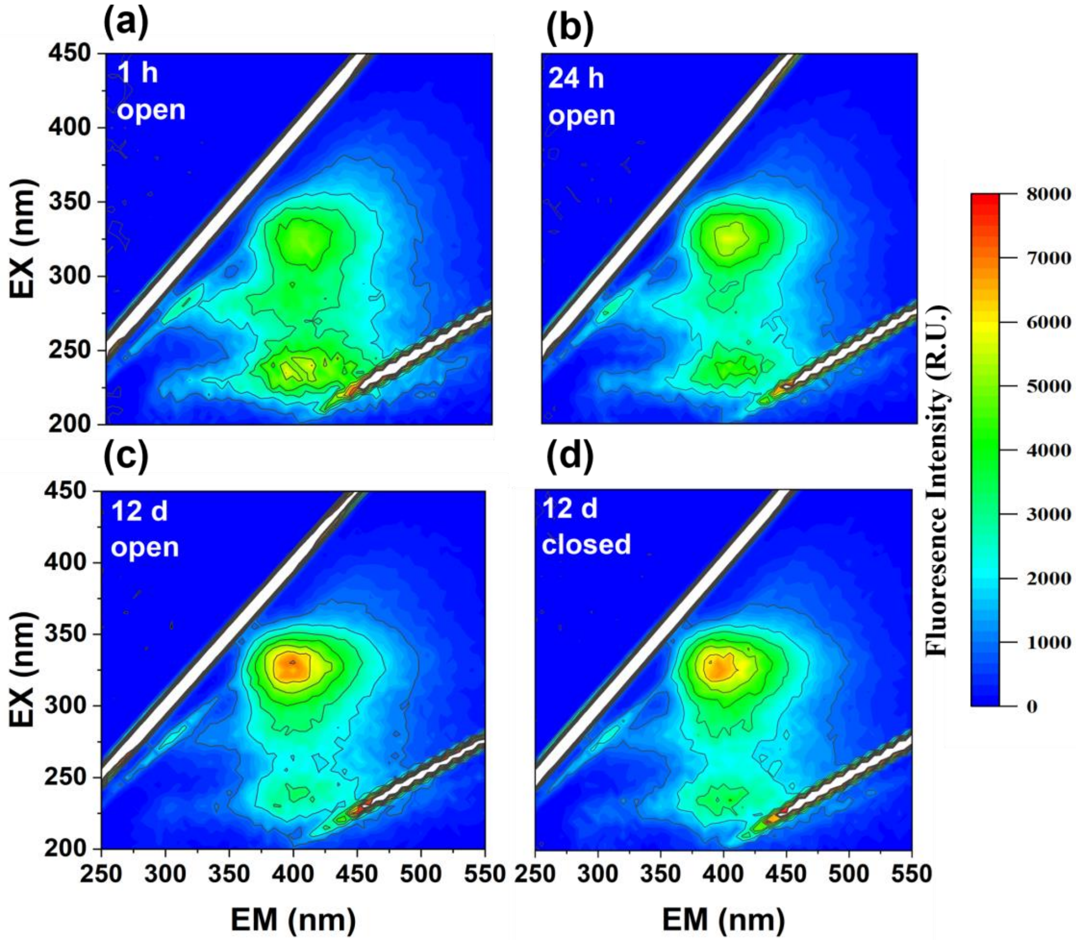

Fig. S11 3D-EEM variation of the product within 12 d. Humification conditions: $[PS]_0$=80 g/L, $[KOH]_0$=80 g/L, ambient temperature=22±1 ^{o}C, reaction time=1 h.

Table S1 Percentage fluorescence response ($P_{region\ number}$) in 3D-EEM five regions of WM under different humification conditions based on FRI method.

Figure No.	P_{I} (%)	P_{II} (%)	P_{III} (%)	P_{IV} (%)	P_{V} (%)
a	18.9±0. 08	37.5±0. 26	17.2±0. 19	18.0±0. 04	8.4±0. 11
b	8.0±0. 11	40.7±0. 24	10.0±0. 08	25.1±0. 13	16.2±0. 15
c	9.4±0.06	28.7±0.04	24.6±0.11	17.6±0.08	19.7±0.16
d	8.9±0. 24	17.9±0. 15	29.2±0. 18	21.3±0. 12	22.7±0. 21
e	5.3±0. 06	18.5±0. 12	28.9±0. 16	15.6±0. 11	31.6±0. 19
f	5.9±0. 11	20.8±0. 08	23.9±0. 13	18.5±0. 16	30.9±0. 09
g	11.2±0. 14	22.6±0. 09	24.5±0. 12	22.8±0. 18	18.9±0. 20
h	4.8±0.08	20.8±0.11	24.5±0.06	16.9±0.13	33.1±0.09
i	20.0±0. 15	41.8±0. 22	15.8±0. 13	15.9±0. 27	6.5±0. 09
j	5.7±0.15	21.8±0.11	29.7±0.08	15.0±0.11	27.8±0.07
k	6.4±0. 12	21.6±0. 17	27.6±0. 13	16.2±0. 08	28.2±0. 32
l	5.3±0. 05	18.5±0. 25	28.9±0. 18	15.6±0. 12	31.7±0. 21
m	5.1±0. 06	18.5±0. 14	29.2±0. 12	15.1±0. 22	32.1±0. 13
n	5.0±0. 11	19.3±0. 22	29.5±0. 08	14.9±0. 13	32.3±0. 29
o	5.1±0. 05	19.2±0. 35	29.2±0. 13	14.1±0. 21	32.4±0. 12
p	5.7±0. 10	20.9±0. 19	29.9±0. 21	15.0±0. 13	32.5±0. 17

Note: error bars represent the standard deviations from triplicate tests.

Table S2 Determination of FLA, SHA, and HLA contents according to eqs. (6-9)

Measured weight (g/50 mL WM)			Calculated weight (g/50 mL WM)	
Product	m_1[a]	HLA	SHA	FLA
13.24±0.1	2.21±0.05	2.5±0.04	5.87±0.09	3.37±0.06

[a]m_1 referred to the dry weight of separated sediment in product at pH 10.5.

Note: m_2 in eq. (6) was calculated to be 5.16 g by assuming complete decomposition of PS. Error bars represent the standard deviations from triplicate tests. SHA and FLA calculated included soluble humic substance either in acid or potassium form.

Table S3 Total organic carbon variation during humification

Total organic carbon (g C/50 mL WM)		Total carbon loss (%)
WM	Product	
4.4±0.2	3.5±0.2	20.5±0.6

Note: data were expressed as mean value ± standard deviation, and the standard deviations were calculated from triplicate tests.

Table S4 Soil physicochemical indexes in different groups

Group	pH	Total N (g/kg)	Total P (g/kg)	Total K (g/kg)	SOM (g/kg)	Available K (mg/kg)	Available P (mg/kg)	Available N (mg/kg)
WM	5±0.2	0.85±0.05a	0.46±0.05a	22.1±0.13b	8.81±0.03a	96.2±0.15d	22.3±0.31c	45.1±0.37c
Blank	5±0.2	0.72±0.08a	0.41±0.03b	21.8±0.11b	8.73±0.05a	96.12±0.26c	21.3±0.15c	44.1±0.22a
ATP	7.4±0.3	0.82±0.02a	0.4±0.03b	21.9±0.02b	8.32±0.15b	97.3±0.16b	22.1±0.26b	45.2±0.51b
K_2SO_4	5.4±0.1	0.81±0.07a	0.41±0.08b	24.2±0.09b	8.26±0.18b	107.7±0.54b	24.2±0.39b	44.6±0.54b
COF	5.9±0.2	0.86±0.06a	0.49±0.06a	23.3±0.23a	8.97±0.07c	123.0±0.23a	24.6±0.18a	43.7±0.12d
Product	7.3±0.4	0.89±0.05a	0.54±0.09a	25.9±0.15a	8.68±0.08c	232.0±0.37a	25.1±0.13a	43.4±0.11d
SRNFF	7.1±0.3	0.87±0.07a	0.56±0.12a	26.2±0.14a	8.64±0.09c	237.0±0.23a	25.8±0.19a	42.8±0.11d

Note: error bars represent the standard deviations from triplicate tests. Comparison among results of different treatments in each column were via one-way ANOVA analysis, and different letters refer to significant differences with Tukey's t test at $p<0.05$ (n=3).

Table S5 Brief comparison of carbon emission and energy consumption between this technology with conventional composting

Items (per ton food waste)	This work	Conventional composting	Saving rate (%)
Carbon loss (%)	20.5	54[1]	62
Electricity consumption (kWh)	6.7	6.7	0
Heat released for reuse (kJ)	8.4	0[a]	100

[a] Too long duration to collect and reuse.

Note S1 Description of different kinetic models

Three kinetic models were used to illustrate the release mechanism of FLA from SRNFF including First-order, Ritger-peppas, and Parabolic Diffusion according to eqs. (1)-(3) [2]:

$$\ln(1-\mathrm{RR}_t)=-kt \tag{1}$$

$$\mathrm{RR}_t=kt^n \tag{2}$$

$$\frac{\mathrm{RR}_t}{t}=kt^{0.5}+b \tag{3}$$

where RR_t(%) referred to the RR of the FLA at time t, k was the kinetic constant related to structural and geometric characteristics of release system, b was a constant, and n was release exponent of the release mechanism: Fickian diffusion (n<0.43) and non-Fickian or anomalous diffusion (0.43<n<1.0).

Note S2 Quantitative analysis of 3D-EEM results

Fluorescence regional integration (FRI) was applied as a quantitative technique for analysis of EEM results. It integrates the volume beneath an EEM. EEM spectra were delineated into five excitation-emission regions according to defined regional boundaries. Volumetric integration under the EEM within each region, normalized to the projected excitation-emission area within that region, resulted in a normalized region-specific EEM volume ($P_{i,n}$) [3]. Note that Milli-Q water blank EEMs were deducted from the sample EEM to decrease the influence of the Rayleigh and Raman scattering.

Note S3 Maillard reaction evidences according to color change, EEM and UV spectra

EEM fluorescence spectroscopy had been widely applied for characterizing the formation of Maillard products at Ex/Em of 340-370 nm/420-440 nm [4], which was consistent with the characteristic EEM spectra in our product (Fig. 1n). According to Fig. 1(i-p), the characteristic fluorescence intensity was gradually strengthened in the first 60 min, which also evidenced the occurrence of Maillard reaction.

Besides, the UV absorbance at 420 nm (A_{420}) had been used as an indicator of the Maillard reaction to show the browning degree [5]. As shown in Fig. S2, A_{420} exhibited a similar trend to the EEM spectra, also suggesting the occurrence of Maillard reaction in the first 60 min.

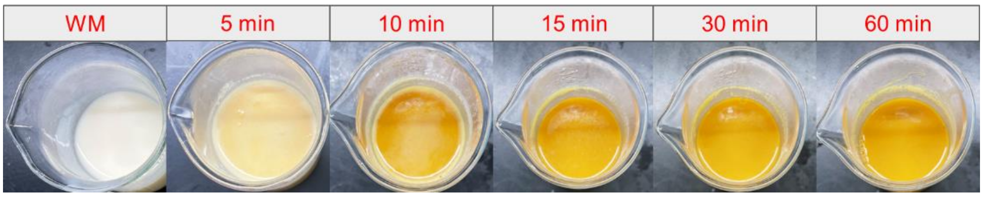

Fig. S12 Color changes of the system during 60 min humification of 50 mL WM by 4 g KOH/ 4 g PS

Text S4 Moisture, FLA and SHA loading calculations of SRNFF

① Dry weight of SRNFF (m_{SRNFF}^{dry}) using 50 mL product, 25 g ATP and 0.1 g XG:

$$m_{SRNFF}^{dry} = m_{product}^{dry} + m_{ATP} + m_{XG} = 13.24\ \text{g} + 25\ \text{g} + 0.1\ \text{g} = 38.34\ \text{g}$$

② Moisture of SRNFF:

$$\text{Moisture\%} = 1 - m_{SRNFF}^{dry}/m_{SRNFF} = 1 - {}^{38.34\ g}/_{55\ g} = 30.3\%$$

③ Content of FLA in SRNFF:

$$\text{FLA\%} = m_{FLA}^{dry}/m_{SRNFF} = {}^{3.37\ g}/_{55\ g} = 6.13\%$$

④ Total amount of FLA for release in 30 g SRNFF:

$$M_{FLA} = 30\ g \times 6.13\% = 183.9\ g$$

⑤ Content of SHA in SRNFF:

$$\text{SHA\%} = m_{SHA}^{dry}/m_{SRNFF} = {}^{5.87\ g}/_{55\ g} = 10.67\%$$

⑥ Total amount of SHA in 30 g SRNFF available for release at pH 10.5:

$$M_{SHA} = 30\ g \times 10.67\% = 3.2\ g$$

Besides, considering the total amount of SHA available for release varied with system pH, accurate amounts of SHA at varied pHs of 3-9 were obtained via eq. (6) by determining corresponding m_1 gravimetrically. The detailed data could be seen in Table S7.

Table S6 m_1 and SHA amounts at varied pHs.

pH	m_1 (g)	SHA in 50 mL product (g)	SHA in 30 g SRNFF (g)
3	3.8	4.3	2.3
4	3.7	4.4	2.4
5	3.6	4.4	2.4
6	3.4	4.7	2.6
7	2.8	5.2	2.9
8	2.6	5.5	3
9	2.5	5.6	3.1
10	2.2	5.9	3.2

References:

[1] Ye, P. *et al.* Insights into carbon loss reduction during aerobic composting of organic solid waste: A meta-analysis and comprehensive literature review. *Science of the Total Environment* **862**, 160787 (2023).

[2] Liu, B. *et al.* Infrared-light-responsive controlled-release pesticide using hollow carbon microspheres@ polyethylene glycol/α-cyclodextrin gel. *Journal of Agricultural and Food Chemistry* **69**, 6981-6988 (2021).

[3] Chen, W., Westerhoff, P., Leenheer J. A., & Booksh K. Fluorescence excitation-emission matrix regional integration to quantify spectra for dissolved organic matter. *Environ. Sci. Technol.* **37**, 5701-5710 (2003).

[4] Wang, Q. *et al.* Mechanistic insights into the effects of biopolymer conversion on macroscopic physical properties of waste activated sludge during hydrothermal treatment: Importance of the Maillard reaction. *Science of the Total Environment* **769**, 144798 (2021).

[5] Xia, X. et al. Formation of fluorescent Maillard reaction intermediates of peptide and glucose during thermal reaction and its mechanism. *J. Agric. Food Chem.* **71**, 8569-8579 (2023).
